# Decreased expression of ARID1A associates with poor prognosis and promotes metastases of hepatocellular carcinoma

**DOI:** 10.1186/s13046-015-0164-3

**Published:** 2015-05-15

**Authors:** Fei He, Jie Li, JianFeng Xu, Sheng Zhang, YaPing Xu, WenXiu Zhao, ZhenYu Yin, XiaoMin Wang

**Affiliations:** Fujian Provincial Key Laboratory of Chronic Liver Disease and Hepatocellular Carcinoma (based on Xiamen University), Xiamen, Fujian China; Department of Hepatobiliary Surgery, Xiamen University Affiliated Zhong Shan Hospital, Xiamen, China

**Keywords:** Hepatocellular carcinoma, ARID1A, Poor prognosis, Migration, Invasion, Metastases

## Abstract

**Background:**

Hepatocellular carcinoma (HCC) is a common malignancy worldwide, which is especially prevalent in Asia. Elucidating the molecular basis of HCC is crucial to develop targeted diagnostic tools and novel therapies. Recent studies have identified AT-rich interactive domain-containing protein 1A (ARID1A) as a broad-spectrum tumor suppressor. We evaluated the clinical implications of decreased *ARID1A* expression in HCC, and investigated the mechanisms of ARID1A*-*mediated tumor suppression.

**Methods:**

Quantitative PCR, western blotting, immunohistochemical analysis of ARID1A mRNA and protein expression was conducted in 64 paired HCC and adjacent non-tumorous tissues. ARID1A function was evaluated *in vitro* in MHCC-97H and Huh7 HCC cell lines, and *in vivo* in a xenografted HCC tumor model.

**Results:**

ARID1A mRNA and protein expression were significantly decreased in HCC tissues, and decreased expression was significantly associated with overall metastasis, including local lymph node and distant metastasis, and poor prognosis. *ARID1A* knockdown promoted HCC cell migration and invasion in vitro, whereas overexpression of *ARID1A* inhibited migration and invasion. E-cadherin levels were closely correlated with ARID1A expression, suggesting a role in migration and invasion. In addition, ARID1A and E-cadherin (*CDH1*) expression were found to be regulated in a coordinated fashion in HCC samples. Furthermore, *ARID1A* knockdown significantly increased HCC tumor growth and lung metastasis *in vivo*.

**Conclusions:**

ARID1A served as an important tumor suppressor. Decreased expression of ARID1A was associated with tumor progression, metastasis, and reduced overall survival in mice and humans. ARID1A could represent a promising candidate therapeutic target for HCC.

**Electronic supplementary material:**

The online version of this article (doi:10.1186/s13046-015-0164-3) contains supplementary material, which is available to authorized users.

## Background

Hepatocellular carcinoma (HCC) is one of the most common malignancies worldwide, and is especially prevalent in Asia [1]. Existing therapies are insufficient for complete tumor eradication. Elucidating the molecular basis of HCC is crucial to develop targeted diagnostic tools and therapies [2]. Genetic lesions play a major role in HCC tumorigenesis and progression. Recently, cancer genome sequencing has identified frequent mutations in epigenetic regulators, particularly chromatin remodeling proteins and histone modifiers, and aberrant chromatin regulation has emerged as a distinct mechanism that contributes to tumor development [3]. Genes encoding subunits of ATP-dependent chromatin remodelers, especially subunits of the SWItch/Sucrose NonFermentable (SWI/SNF) complex, are frequently mutated in a broad array of cancer types [4].

AT-rich interactive domain-containing protein 1A (ARID1A) is a key member of the SWI/SNF chromatin-remodeling complex. Also known as BAF250a, SMARCF1, or p270, ARID1A belongs to a family of proteins that contain a highly conserved, ~100 amino acid DNA binding domain termed ARID (AT-rich interacting domain) [5]. ARID1A has been implicated in numerous protein-protein interactions, and the most widely known and studied are those which make ARID1A a part of SWI/SNF chromatin remodeling complexes. As a member of SWI/SNF complexes, ARID1A is thought to contribute to specific recruitment of its chromatin remodeling activity by binding transcription factors and transcriptional coactivator/corepressor complexes [6, 7].

Several genome-wide sequencing studies have uncovered frequent *ARID1A* mutations in a multitude of human cancers including subtypes of ovarian [8, 9], endometrial [10], uterine cancers [11], gastric carcinoma [12, 13], esophageal adenocarcinoma [14], breast cancer [15] and transitional cell carcinoma of the bladder [16]. In liver cancer, *ARID1A* mutations were observed in 10–16.8 % of the studied tumors [17, 18] and in 13 % of hepatitis B virus-associated hepatocellular carcinomas [19]. Furthermore, many *ARID1A* mutations are insertion/deletion mutations, leading to downregulation of the encoded protein [20, 21]. Immunohistochemistry assays demonstrated that a substantial proportion of uterine endometrioid carcinomas, uterine clear-cell carcinomas, uterine serous carcinomas, and uterine carcinosarcomas also have loss of ARID1A protein (BAF250a) [10]. In two independent cohorts of >200 human breast cancer cases, low ARID1A protein expression was associated with more aggressive breast cancer phenotypes, such as those with a high tumor grade [15]. ARID1A protein loss also correlated with an advanced stage in non-small cell lung cancer [22]. However, the clinical significance of ARID1A and its biological function in HCC has not yet been clarified. In the present study, we investigated ARID1A protein expression in HCC tissues, and analyzed the correlation between the loss of ARID1A expression and the clinicopathological features of HCC. In addition, we explored the possible mechanisms by which ARID1A affects HCC metastases. Finally, we evaluated the role of ARID1A in HCC cell migration and invasion *in vitro*, and conducted HCC tumor xenograft studies to determine the biological functions of ARID1A *in vivo*.

## Materials and methods

### Tissue samples

Sixty-four patient-derived paired HCC and adjacent nontumorous tissue samples were collected at the Zhongshan Hospital of Xiamen University. Written informed consent was obtained from all patients, and the study was approved by the Clinical Research Ethics Committee of Zhongshan Hospital of Xiamen University.

### RNA extraction and quantitative real-time reverse transcription polymerase chain reaction

Total RNA was isolated using TRIzol® Reagent (#15596-018, Life Technologies, New York, USA) according to manufacturers’ instructions. Subsequently, cDNA was generated using the PrimeScript™ RT reagent Kit with gDNA eraser (Takara Bio Inc., Dalian, China), and quantitative real-time reverse transcription polymerase chain reaction (qPCR) was performed using the Real-Time PCR detection system (#7500, Applied Biosystems, Shanghai, China) with 2× SYBR Green II/ROX qPCR Master Mix (Takara Bio Inc.). Relative mRNA expression was calculated using the delta threshold cycle (ΔΔCT) method and normalized to β-actin (*ACTB*) expression. The PCR primers are listed in Additional file 1: Table S1.

### Immunohistochemical analysis of patient-derived HCC tissues

Surgically excised tumor specimens were fixed in 10 % neutral formalin and embedded in paraffin, and 4-μm thick sections were prepared using a microtome (#HM315, Thermo Scientific, Waltham, USA). Free-floating section immunostaining was performed using the avidin–biotin–peroxidase complex method (UltraSensitive™, Maixin, Fuzhou, China). Sections were deparaffinized in xylene, and rehydrated in a graded ethanol series. They were then placed in EDTA antigen retrieval buffer (pH 9.0, MVS-0099 Maixin, Fuzhou, China) and incubated at 121 °C for 3 min in an autoclave. Endogenous peroxidase activity was blocked by placing the specimens in 3 % hydrogen peroxide solution for 10 min. Sections were incubated overnight at 4 °C in an anti-human ARID1A antibody (rabbit polyclonal, 1:500, HPA005456, Sigma, USA). Sections were stained in parallel with non-immune immunoglobulin G as a negative control. Antibody binding was detected using an Elivision plus kit (Elivision™ super KIT9922, Maixin, Fuzhou, China), which uses 3, 3’-diaminobenzidine for visualization. Sections were counterstained with hematoxylin, then dehydrated, and coverslips were mounted onto slide-fixed specimens for microscopy. Slides were examined by 2 investigators in an independent and random manner. Five views per slide with 100 cells/view were evaluated at 400× magnification using a light microscope (#Axio Scope A1 pol, Carl Zeiss, Germany). Nuclear staining was considered as positive. Immunohistochemical grading was performed using the following scoring system: 0, 0–10 %; 1, 11–29 %; 2, 30–59 %; and 3, > 60 %. Samples had a higher score in HCC than in notumor tissue were defined as ARID1A high. Otherwise, they were be defined as ARID1A low. Hematoxylin-eosin (HE) stain was performed as previous described [23].

### Clonal cell culture and small-interfering RNA

A panel of human HCC cell lines including MHCC-97H, LM3, SK-Hep1, SMMC-7721, HepG2, and Huh7 were purchased from the Cell Bank of Shanghai, Institutes for Biological Sciences, China. HEK293T and GP2-293 cells were generously gifted by the Medical College of Xiamen University. All clonal cells except SMMC-7721 cells were cultured in Dulbecco’s Modified Eagle’s Medium supplemented with 10 % fetal bovine serum in a humidified incubator at 37 °C and 5 % CO_2_. The short hairpin RNA (shRNA) retroviral plasmid (RNAi-Ready pSIREN-RetroQ), which contains a puromycin resistance gene, was purchased from Clontech. The ARID1A shRNA sequences cloned into this vector are shown in Additional file 1 Table S2. The full coding sequence of ARID1A was cloned into the lentiviral pLV-CS2.0 vector that contains an EF1α promoter to drive expression. All transfections were performed using Turbofect Transfection Reagent (#R0531, Thermo Scientific, Waltham, USA). Puromycin was used to generate cells with stable knockdown of ARID1A.

### Western blotting

Total protein was extracted from cells in RIPA lysis buffer (#P0013B, Beyotime, Shanghai, China) and quantified using a Bradford assay. In total, 30 μg of protein was separated using 10 % sodium dodecyl sulfate-polyacrylamide gel electrophoresis (SDS-PAGE) and then transferred to a polyvinylidene difluoride (PVDF) membrane (Millipore, Bedford, MA, USA). The membrane was blocked in a 5 % powdered milk solution and incubated in primary antibody overnight at 4 °C. After washing, the membrane was incubated with a horseradish peroxidase–conjugated secondary antibody (Santa Cruz Biotechnology, Dallas, USA.) at 37 °C for 1 h. Protein bands were visualized using Western Bright ECL (#K-12049-D50, Advansta, CA, USA) and detected using ImageQuant LAS4000mini (General Electric, USA). Relative protein levels were calculated based on a glyceraldehyde 3-phosphate dehydrogenase (GAPDH) loading control. Antibodies used included E-cadherin (#3915S, Cell Signaling technology, Boston, USA), Vimentin (#5741S, Cell Signaling technology), Fibronectin (#610077, BD Bioscience, Bedford, USA), GAPDH (#AB-M-M 001, Hangzhou Xianzhi Biotechnology, Hangzhou, China), and ARID1A (#04-080, Millipore, Shanghai, China).

### Cell migration and invasion assays

Migration of HCC cells was assessed using the 24-well polycarbonate membrane cell migration assay kit (#3422, Corning Incorporated Costar, Tewksbury, USA) according to the manufacturer’s instructions. Briefly, HCC cell lines were transfected with control and ARID1A shRNAs and were incubated in serum-free medium for 24 h. The cells were then transferred to the upper chamber of a Transwell plate by seeding 2 × 10^5^ cells per well in 200 μL serum-free medium. Next, 0.5 mL of 10 % fetal bovine serum-containing medium was added to the lower chamber as a chemoattractant. Cells were incubated for 24–48 h (depending upon migration capability) at 37 °C. Non-migrating cells on the upper membrane surface were scraped off using cotton swabs. Cells that migrated to the bottom of the membrane were stained with 0.1 % crystal violet for 30 min, followed by washing with water for 30 s to remove residual dye. Four views were examined per transwell and cells/view were counted at 200× magnification. Each experiment was performed in triplicate. The invasion assay was performed in a similar fashion using BD BioCoat™ Matrigel™ Invasion Chambers (#354480, BD Biosciences), except that the upper chambers were precoated with ECMatrix™ gel.

### Cell viability assay

Cell growth was determined using a Cell Counting Kit-8 (CCK-8) cell viability assay (#YB-K001, Yiyuan Biotechnologies, Guangzhou, China) according to the manufacturer’s instructions as described previously [24].

### Cell apoptosis detection

Cell apoptosis detection was performed with Annexin V/PI (propidiumiodide) double staining (#KGA108KeyGEN Biotech, Nanjing, China). Briefly, 48 h after transfection and 12 h Cisplatin (20 μM) treatment, cells were harvested by 0.25 % trypsin (without EDTA), washed twice with chilled PBS, followed by resuspension in 200 μL of binding buffer. Staining solution containing Annexin V/FITC and PI was added to the cell suspension. After incubation in the dark for 30 min, the cells were analyzed by FACS Gallios flow cytometer (Beckman Coulter, USA).

### Analysis of ARID1A function in HCC tumor xenografted mice

All animal protocols were approved by the Animal Care and Use Committee of Xiamen University. We purchased male BALB/c nude mice (4–5weeks old) from Shanghai Experimental Animal Center of Chinese Academic of Sciences (Shanghai, China). Animals were kept under standard pathogen-free conditions and allowed to acclimate for 1 week before use. MHCC-97H cells (5 × 10^6^/0.2 mL of PBS) stably transfected with either control or ARID1A shRNA expression vectors were subcutaneously injected into right flank of each mouse (n =8 mice/group). Tumor growth was monitored once a week using a caliper, and the tumor volume was calculated using the following formula: volume = π/6 × length × width^2^. We monitored tumor growth over an 8-week period.

### Statistical analyses

The statistical package SPSS 19.0 (SPSS, Chicago, IL, USA) was used for all analyses. All values are expressed as mean ± SEM. Correlations of ARID1A expression with clinicopathological characteristics were evaluated with a *χ*^2^ test using R language. Survival analyses were conducted using the Kaplan-Meier method with the log-rank test. Other results were analyzed using a Student’s *t* test. All p-values were two-sided, and p <0.05 indicated statistical significance

## Results

### Reduced expression of ARID1A in HCC patients was associated with poor prognosis and an increased risk of metastasis

**Fig. 1:**
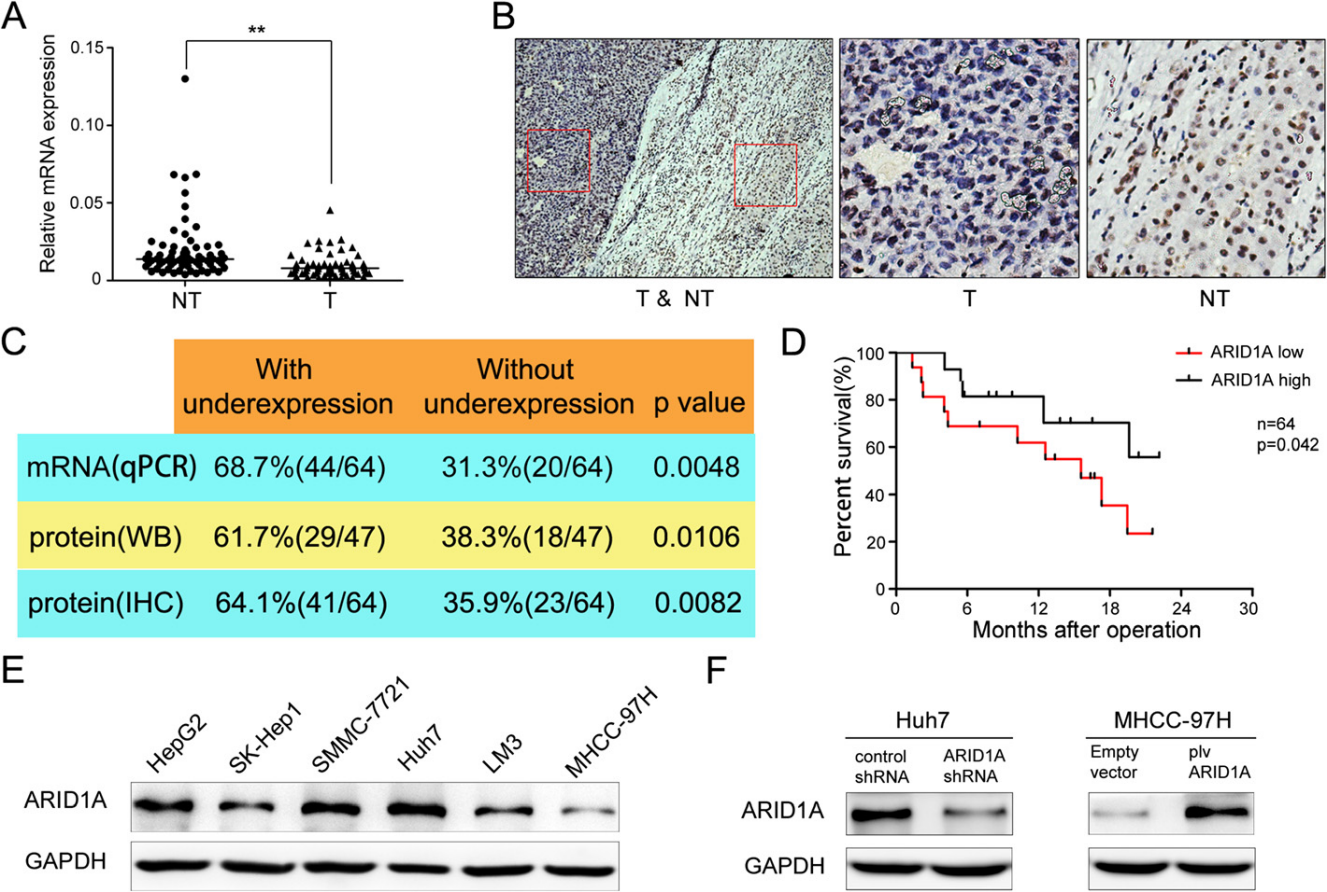
Recurrent reduction of *ARID1A* expression in hepatocellular carcinoma was associated with poor prognosis. **a** mRNA level of *ARID1A* was quantified with qPCR in 64 paired HCC (T) and nontumorous tissues (NT). Horizontal lines indicate the median of biological replicates. Significant differences between normal and cancer tissue were calculated by Student’s *t*-test (p < 0.01). **b** Immunohistochemistry of ARID1A (BAF250a) protein expression in HCC surgical specimens. Strong ARID1A-positive staining in normal tissue is shown in the right panel, whereas ARID1A-negative staining in HCC is presented in the middle panel. Red boxes in the left panel indicate the area enlarged in the middle and right panels. **c** The summary of the differences in the expression of ARID1A protein and mRNA between paired tumor and non-tumor liver tissues (right panel). **d** Kaplan–Meier survival curves of HCC patients (n = 64, all from Zhongshan Hospital of Xiamen University) after hepatectomy. Patients with low ARID1A expression had a significantly worse prognosis than those with high ARID1A expression (log-rank test, p = 0.042). **e** Endogenous expression of ARID1A was examined in HepG2, SK-Hep1, SMMC-7721, Huh7, LM3 and MHCC-97H cell lines by western blot. **f** Western blot analysis showed that *ARID1A* shRNA treatment markedly decreases its protein levels in Huh7 cells while plv cs2.0 plasmid transfection significantly enhanced ARID1A expression in MHCC-97H cells

As revealed by qPCR, *ARID1A* mRNA levels were significantly downregulated in HCC tissues compared with nontumorous tissues (n = 64, Student’s *t*-test, p <0.01) (Fig. 1a). In the same 64 paired HCC samples, immunohistochemical analysis revealed that ARID1A protein expression was decreased in 41 out of 64 (64.1 %) tumor tissues when compared with adjacent non-tumorous counterparts. And 36 out of the 41 ARID1A protein downregulated samples also had reduced ARID1A mRNA expression. Strong ARID1A-positive nuclear staining was found in normal tissues (Fig. 1b, right panel), whereas ARID1A-negative staining (dark blue) can be seen in tumor tissues (Fig. 1b, the middle panel). Western blotting quantification of ARID1A was consistent with the immunohistochemical data, in which ARID1A expression in 61.7 %(29/47) HCC tissues was significantly lower than their adjacent nontumorous tissues counterparts (Fig. 1c and Additional file 2: Figure S1).

After surgical resection, patients with tumors with low ARID1A expression showed a significantly worse prognosis compared with those with high ARID1A expression (log-rank test, n = 64, p = 0.042) (Fig. 1d). In addition, low ARID1A expression in tumors was significantly correlated with a higher metastatic rate including local lymph node and distant metastases (Additional file 1: Table S3). Univariate analyses of the 64 paired HCC cases indicated that there was no significant difference in ARID1A expression according to age, sex, liver cirrhosis, hepatitis virus B infection, or serum alfa-fetoprotein levels.

### ARID1A knockdown promotes HCC cell migration and invasion, whereas overexpression of ARID1A inhibits HCC cell migration and invasion

In order to investigate the function of ARID1A *in vitro*, we used a panel of HCC cell lines. Western blot analysis indicated relatively high ARID1A expression in Huh7 cells, whereas MHCC-97H cells lacked ARID1A expression (Fig. 1e), so these cell lines were chosen for subsequent knockdown and overexpression analyses, respectively. The efficiencies of knockdown and overexpression in these cells are shown in Fig. 1f.

**Fig. 2:**
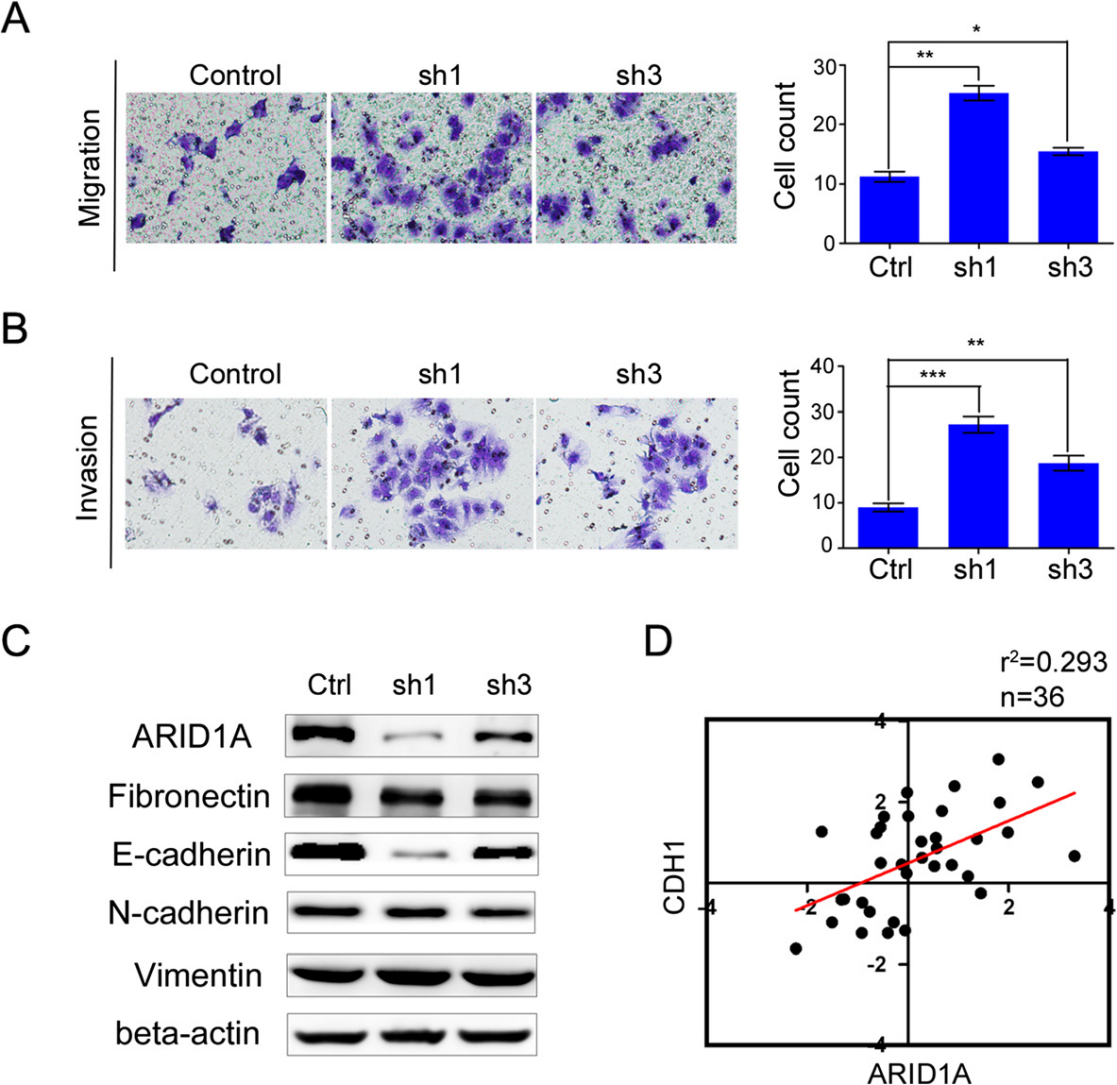
Silencing of *ARID1A* promotes HCC cell migration and invasion. **a** shows cell migration ability was increased in *ARID1A* knockdown Huh7 cell line, while (**b**) exhibits its effect on invasion. For migration/invasion assay cells were cultured in the upper chamber of transwell for 24 h/48 h respectively. **c** We examined epithelial–mesenchymal transition (EMT) associated proteins, including E-Cadherin, N-Cadherin, Fibronectin and Vimentin in *ARID1A* knockdown Huh7 cells. **d** The expression level of both *ARID1A* and *CDH1* (E-Cadherin) was evaluated by qPCR in 36 paired HCC specimens collected from Zhongshan Hospital, Xiamen, China. Correlation coefficient (*R* ^2^) of both proteins was calculated as log2-transformed expression between normal and cancer tissues

**Fig. 3:**
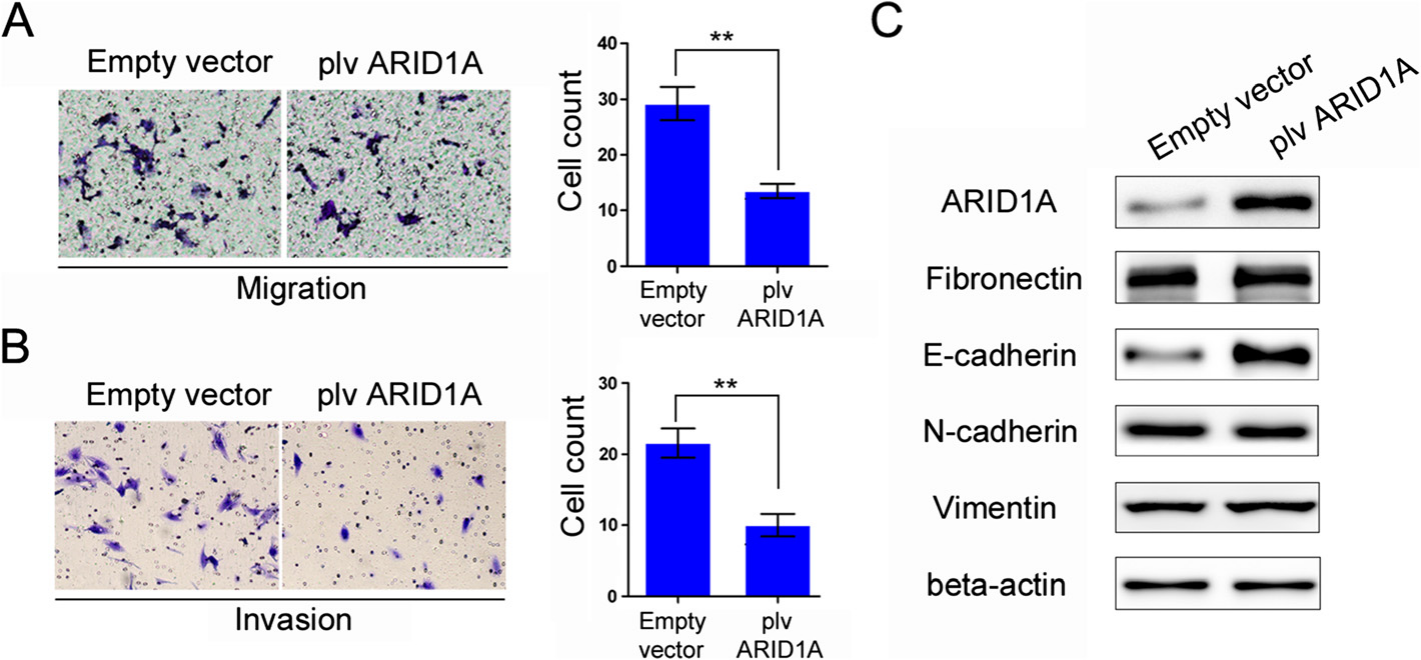
Overexpression of *ARID1A* inhibits HCC cells migration and invasion. **a** Left panel shows cell migration ability was decreased in *ARID1A* overexpressed MHCC-97H cells, right panel exhibits statistical description of result A. **b** Cell invasive capacity was inhibited in MHCC-97H cells with forced expression of *ARID1A*. **c** Western blot analysis showed that *ARID1A* transfection increased E-cadherin expression, but Vimentin was not significantly affected

shRNA-mediated knockdown of *ARID1A* significantly promoted migration and invasion of Huh7 cells (sh1 = 124.40 % and sh3 = 37.7 %; and sh1 = 200.2 %, and sh3 = 100.8 %, respectively; Fig. 2a-b). To investigate the molecular mechanism underlying the role of ARID1A in migration and invasion, we investigated the association of ARID1A with proteins that regulate epithelial–mesenchymal transition. E-cadherin was significantly downregulated in *ARID1A*-silenced cells (Fig. 2c), whereas Fibronectin, N-cadherin and Vimentin expressions were not significantly affected (Fig. 2c). In addition, there was a strong correlation between ARID1A and E-cadherin (*CDH1*) expression in HCC tissue samples (Fig. 2d). Conversely, overexpression of *ARID1A* in MHCC-97H cells significantly inhibited migration and invasion (Fig. 3a-b). Furthermore, E-cadherin expression was significantly increased in ARID1A overexpressing cells (Fig. 3c).

### ARID1A impaired in vivo xenograft tumor growth and HCC lung metastasis

**Fig. 4:**
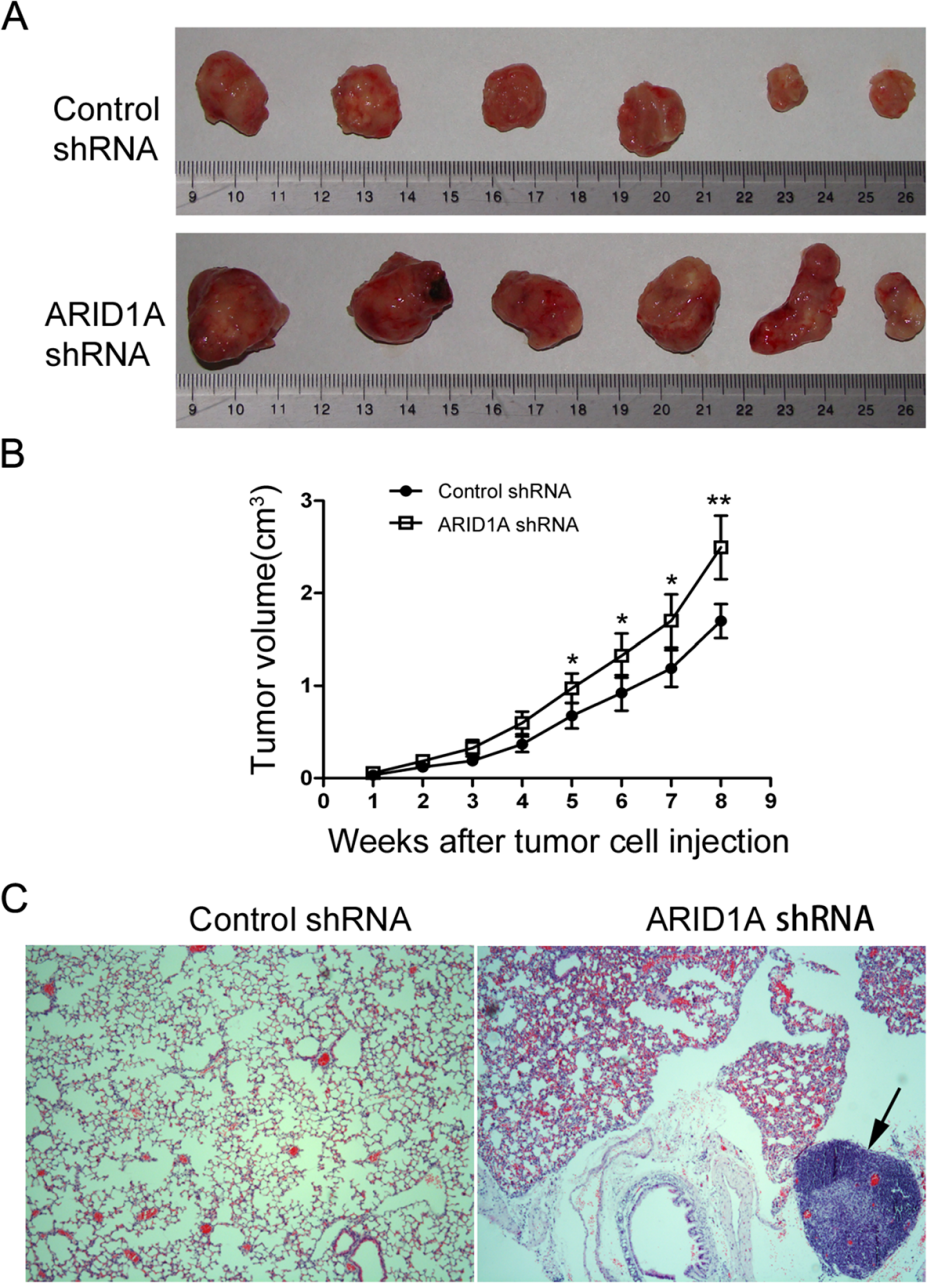
Downregulation of *ARID1A* increases *in vivo* xenograft tumor growth. **a** Images of xenografts in *ARID1A* knockdown group (*bottom*) and control group (*top*) at the end of the experiment. Control shRNA and *ARID1A* shRNA cells were injected subcutaneously into nude mice (n = 8 for each group), and the tumors were isolated 8 weeks later. **b** *ARID1A* knockdown promotes xenograft growth of MHCC-97H cells in nude mice (n = 6, P < 0.005). **c** Silencing *ARID1A* in HCC cells promotes its lung metastasis, left panel shows the HE image of mouse lung tissue in control group, right panel exhibits the HE image of mouse lung tissue with HCC metastasis, indicated by the black arrow

In addition to examine the biological functions of *ARID1A in vivo*, we also assessed the functions of *ARID1A* by using a xenograft transplantation model in nude mice. We subcutaneously transplanted the same amount of Control shRNA and *ARID1A* shRNA cells into nude mice respectively; thereafter we monitored the tumor growth over a 8-week period. As shown in Fig. 4a, 8 weeks after transplantation, tumor growth in *ARID1A* knockdown transplanted mice was significantly greater compared with that in control-shRNA transplanted mice (p < 0.01). Our results exhibited the tumour growth curve on the time course after tumor cell injection (Fig. 4.b), and the difference between Control group and kockdown group can easily be witnessed. Three out of 6 *ARID1A* knockdown mice developed lung metastasis of HCC (Fig. 4c), whereas none of the mice in the control shRNA group showed any lung metastases.

## Discussion

The *ARID1A* gene has been classified as a novel tumor suppressor, as evidenced by associations between ARID1A mRNA or protein expression and several cancers including ovarian, endometrial, gastric, and breast cancers [10, 25]. Based on the results of a previous whole-exome sequencing study [26], we evaluated the comprehensive role of ARID1A in HCC.

Reduced *ARID1A* expression was associated with lymph node metastasis, tumor infiltration, and poor prognosis in patients with gastric carcinoma [27, 28]. Similarly, the present study found that ARID1A protein expression was decreased in patient-derived HCC tumor tissues, and that decreased expression was significantly correlated with lymph node and distant metastasis, and poor prognosis.

Previous studies demonstrated that ARID1A also served as a regulator of cell proliferation and survival [22, 29]. So, we checked its role in HCC cell proliferation and apoptosis as well. Here, *ARID1A* knockdown promoted HCC cell proliferation (Additional file 3: Figure S2-A), while overexpression of *ARID1A* inhibited proliferation and impaired clonal formation in HCC cells (Additional file 3: Figure S2-B). These results are consistant with the previous findings that Yi Zhang.*et al.* did in non-small cell lung cancer [22]. We also evaluated a putative role of ARID1A in mediating cisplatin-induced apoptosis in HCC cell lines, and found that overexpression of *ARID1A* promoted cisplatin-induced apoptosis (Additional file 3: Figure S2-C). This was congruent with the findings from a previous study that evaluated decreased ARID1A expression in a leukemia cell line with conferred resistance to Fas-mediated apoptosis [29].

Since epithelial-mesenchymal transition (EMT) is one of the crucial events regulating hepatocellular carcinoma, prostate cancer invasion and metastasis [30, 31], we checked EMT associated proteins in *ARID1A* knockdown and overexpression cells. In our study, the expression of E-cadherin was strongly correlated with that of ARID1A, suggesting that the two interact in some way to regulate migration and invasion. E-cadherin is a core protein mediated cell-cell adhesion to hold the epithelial cells tight together. Loss of E-cadherin decreases the cellular adhesion, resulting in an increase of cell motility [32]. However, *ARID1A* silencing did not induce epithelial–mesenchymal transition in HCC cells, as evidenced by a lack of any changes in cell morphology in HCC cell lines subjected to *ARID1A* knockdown. Considering that E-cadherin is essential for cell adhesion, it is possible that decreased ARID1A expression in HCC tissues might loosen cell-cell junctions, promoting the migration and invasive capacity of tumor cells. Additional studies are needed to elucidate how ARID1A interacts with E-cadherin in HCC.

In nature, HCC is an invasive tumor that metastasizes hematogenously and lymphogenously to other organs, even after local recurrence. The most common organs of distant metastases include the lungs, lymph nodes, bone, and brain, with the lung metastasis occurring in 18–60 % of HCC cases [33]. This was reflected in the present study with the development of lung metastasis in 50 % of the mice bearing HCC tumors with ARID1A knockdown, further implicating decreased ARID1A expression with the development of metastasis in HCC.

Our results,together with previous mutational and functional studies, suggest ARID1A is a *bona fide* tumor suppressor. Therefore, it will be of interest to determine whether depletion of ARID1A can be therapeutically exploited by targeting downstream and potentially reversible epigenetic consequences of remodeler mutation [34].

## Conclusion

In conclusion, our results have shown that *ARID1A* is frequently downregulated in hepatocellular carcinoma and is related to the aggressive phenotype of HCCs. We explored comprehensive role of the gene in HCC, which might provide direction for future studies on the molecular mechanisms of *ARID1A*. ARID1A represents a potential drug candidate for molecular-targeted therapy for HCC.

## Additional files

Additional file 1: Table S1. Information of qPCR Primers; **Table S2.** ARID1A shRNA sequence list; **Table S3.** Correlation between the clinicopathological characteristics and expression of the ARID1A (BAF250a) protein in hepatocellular carcinoma. Additional file 2: Figure S1. Western blot analysis of ARID1A protein expression in 47 human HCC samples and their adjacent non-tumorous liver tissue samples. N: non-tumorous liver tissue, T: tumor tissue. Additional file 3: Figure S2.
*ARID1A* depletion facilitates cell proliferation and inhibits apoptosis. (A) CCK8 assay in Huh7 cells transfected with ARID1A shRNA. A time dependent increase was witnessed in cell proliferation after ARID1A shRNA transfection compared with control, but *ARID1A* overexpression in MHCC-97H cells caused a decrease in cell proliferation. (B) Colony formation assay was performed in Huh7 cells with *ARID1A* knockdown and MHCC-97H cells with forced expression of *ARID1A*. A marked increase in colony formation is seen in the groups with *ARID1A* depletion. (C) AnnexinV/PI analysis showed that *ARID1A* depletion inhibited early apoptosis induced by Cisplatin, while cells with overexpression of ARID1A are more sensitive to Cisplatin-induced early apoptosis. (* p < 0.05, ** p < 0.01).

## Abbreviations

ARID1A: AT-rich interactive domain-containing protein 1A
BAF250a: BRG1-associated factor 250a
CDH1: Cadherin 1
IHC: Immunohistochemistry
qPCR: Quantitative reverse transcription PCR
shRNA: Short hairpin RNA
SWI/SNF: SWItch/Sucrose non-fermentable
