# Supplementary material for: Decreased expression of ARID1A associates with poor prognosis and promotes metastases of hepatocellular carcinoma
Source: J Exp Clin Cancer Res. 2015 May 15;34(1):47. doi: 10.1186/s13046-015-0164-3 (PMC4440314; doi:10.1186/s13046-015-0164-3)
Supplement: Additional file 1: Table S1. — Information of qPCR Primers; Table S2. ARID1A shRNA sequence list; Table S3. Correlation between the clinicopathological characteristics and expression of the ARID1A (BAF250a) protein in hepatocellular carcinoma. [file 13046_2015_164_MOESM1_ESM.doc]

Supplementary Table 1:Information of qPCR Primers

| gene name | sequence |
| --- | --- |
| ***ARID1A*** | **Forward**  **TCCCAGCAAACTGCCTATTC (Sense)** |
|  | **Reverse**  **CATATCTTCTTGCCCTCCCTTAC (AntiSense)** |
| ***ACTB*** | **Forward**  **TCCCTGGAGAAGAGCTATGA (Sense)** |
|  | **Reverse**  **CAGGAAGGAAGGCTGGAAA (AntiSense)** |
| ***CDH1*** | **Forward**  **TCGACAAAGGACAGCCTATTT (Sense)** |
|  | **Reverse**  **TGTGGGTTATGAAACCGTAGAG (AntiSense)** |

Supplementary Table 2:ARID1A shRNA sequence list.

| name | sequence |
| --- | --- |
| **sh1** | Top Strand (66bp)  5'-gatccGCCCTGAACAATAACCTCACTTCAAGAGAGTGAGGTTATTGTTCAGGGTTTTTTACGCGTg-3' |
|  | Bottom Strand (66bp)  5'-aattcACGCGTAAAAAACCCTGAACAATAACCTCACTCTCTTGAAGTGAGGTTATTGTTCAGGGCg-3' |
| **sh2** | Top Strand (65bp)  5'-gatccAGATGAGACCTCAGCCATATTCAAGAGATATGGCTGAGGTCTCATCTTTTTTTACGCGTg-3' |
|  | Bottom Strand (65bp)  5'-aattcACGCGTAAAAAAAGATGAGACCTCAGCCATATCTCTTGAATATGGCTGAGGTCTCATCTg-3' |
| **sh3** | Strand (66bp)  5'-gatccGCCTCAATGACCTCCAGTAATTCAAGAGATTACTGGAGGTCATTGAGGTTTTTTACGCGTg-3' |
|  | Bottom Strand (66bp)  5'-aattcACGCGTAAAAAACCTCAATGACCTCCAGTAATCTCTTGAATTACTGGAGGTCATTGAGGCg-3' |

Supplementary Table 3

Correlation between the clinicopathological characteristics and expression of the ARID1A(BAF250a) protein in hepatocellular carcinoma.

| Characteristics | Number of | ARID1A | ARID1A | p value |
| --- | --- | --- | --- | --- |
|  | patients | downregulated | positive |  |
| Age |  | | | 0.206 |
| <60 | 36 | 21(32.81%) | 15(23.44%) |  |
| ≥60 | 28 | 20( 31.25%) | 8(12.50%) |  |
| Gender |  | | | 0.554 |
| Female | 48 | 31(48.44%) | 17(26.56%) |  |
| Male | 16 | 10(15.63%) | 6(9.38%) |  |
| Tumor size |  | | | 0.252 |
| <5cm | 34 | 20(31.25%) | 14(21.88%) |  |
| ≥5cm | 30 | 21(32.81%) | 9(14.06%) |  |
| Serum AFP, ng/mL |  | | | 0.488 |
| <400 | 18 | 11(17.19%) | 7(10.94%) |  |
| ≥400 | 46 | 30(46.88%) | 16(25.00%) |  |
| Differentiation |  | | | 0.557 |
| moderate-well | 52 | 33(51.56%) | 19(29.69%) |  |
| poor | 12 | 8(12.50%) | 4(6.25%) |  |
| HBV infection |  | | | 0.159 |
| No | 47 | 32(50.00%) | 15(23.44%) |  |
| Yes | 17 | 8(12.50%) | 9(14.06%) |  |
| Liver cirrhosis |  |  |  | 0.396 |
| No | 41 | 27(42.19%) | 14(21.88%) |  |
| Yes | 23 | 14(21.88%) | 9(14.06%) |  |
| Overall metastasis* |  |  |  | 0.031 |
| No | 39 | 29(45.31%) | 10(15.63%) |  |
| Yes | 25 | 12(18.75%) | 13(20.31%) |  |
| Tumor stage (AJCC) |  |  |  | 0.211 |
| I–II | 40 | 27（42.18%） | 13（20.31%） |  |
| III–IV | 24 | 13（20.31%） | 11（17.18%） |  |

Abbreviation: AFP, alpha-fetoprotein.

*Overall metastasis includes local lymph node metastasis and distant metastasis
