# Supplementary figures and images for: Decreased expression of ARID1A associates with poor prognosis and promotes metastases of hepatocellular carcinoma
Source: J Exp Clin Cancer Res. 2015 May 15;34(1):47. doi: 10.1186/s13046-015-0164-3 (PMC4440314; doi:10.1186/s13046-015-0164-3)

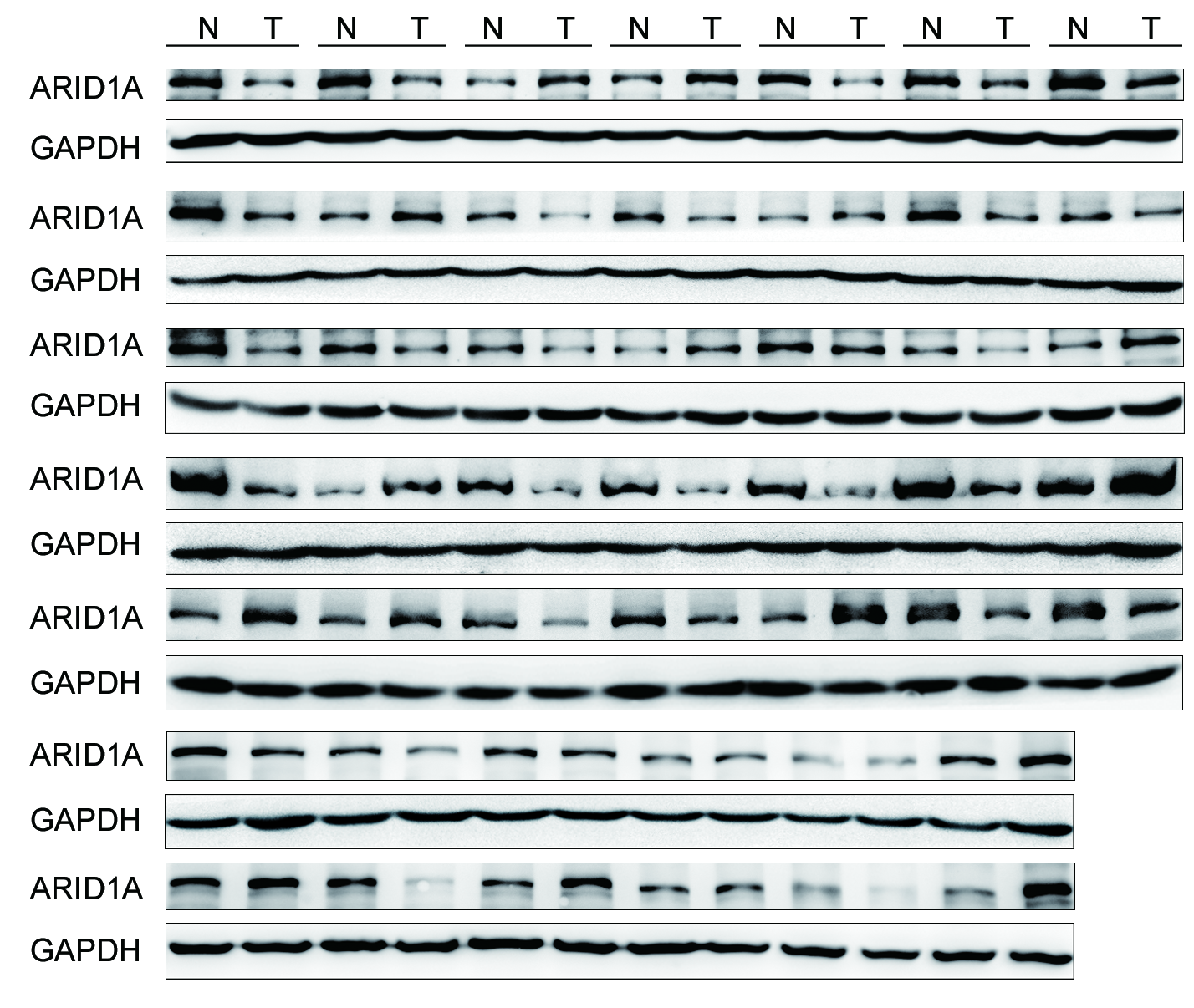

Supplement: Additional file 2: Figure S1. — Western blot analysis of ARID1A protein expression in 47 human HCC samples and their adjacent non-tumorous liver tissue samples. N: non-tumorous liver tissue, T: tumor tissue. [file 13046_2015_164_MOESM2_ESM.tif]

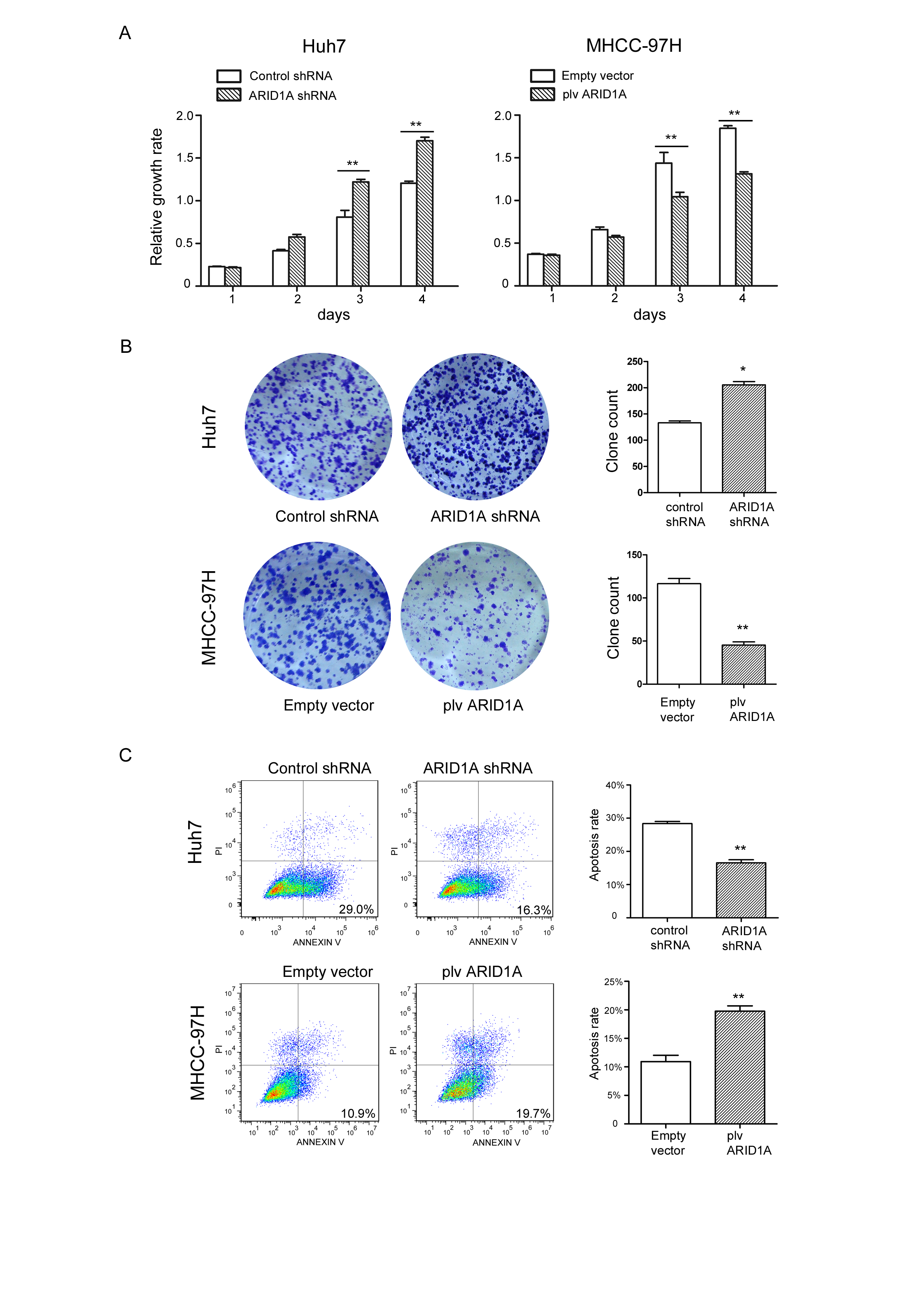

Supplement: Additional file 3: Figure S2. — ARID1A depletion facilitates cell proliferation and inhibits apoptosis. (A) CCK8 assay in Huh7 cells transfected with ARID1A shRNA. A time dependent increase was witnessed in cell proliferation after ARID1A shRNA transfection compared with control, but ARID1A overexpression in MHCC-97H cells caused a decrease in cell proliferation. (B) Colony formation assay was performed in Huh7 cells with ARID1A knockdown and MHCC-97H cells with forced expression of ARID1A. A marked increase in colony formation is seen in the groups with ARID1A depletion. (C) AnnexinV/PI analysis showed that ARID1A depletion inhibited early apoptosis induced by Cisplatin, while cells with overexpression of ARID1A are more sensitive to Cisplatin-induced early apoptosis. (* p < 0.05, ** p < 0.01). [file 13046_2015_164_MOESM3_ESM.tif]
